# Characterization of Schizophrenia Adverse Drug Interactions through a Network Approach and Drug Classification

**DOI:** 10.1155/2013/458989

**Published:** 2013-09-09

**Authors:** Jingchun Sun, Min Zhao, Ayman H. Fanous, Zhongming Zhao

**Affiliations:** ^1^Department of Biomedical Informatics, Vanderbilt University School of Medicine, Nashville, TN 37203, USA; ^2^Center for Quantitative Sciences, Vanderbilt University Medical Center, Nashville, TN 37232, USA; ^3^Mental Health Service Line, Washington VA Medical Center, 50 Irving St. NW, Washington, DC 20422, USA; ^4^Virginia Institute for Psychiatric and Behavioral Genetics, Virginia Commonwealth University, Richmond, VA 23298, USA; ^5^Department of Psychiatry, Vanderbilt University School of Medicine, Nashville, TN 37212, USA; ^6^Department of Cancer Biology, Vanderbilt University School of Medicine, Nashville, TN 37232, USA

## Abstract

Antipsychotic drugs are medications commonly for schizophrenia (SCZ) treatment, which include two groups: typical and atypical. SCZ patients have multiple comorbidities, and the coadministration of drugs is quite common. This may result in adverse drug-drug interactions, which are events that occur when the effect of a drug is altered by the coadministration of another drug. Therefore, it is important to provide a comprehensive view of these interactions for further coadministration improvement. Here, we extracted SCZ drugs and their adverse drug interactions from the DrugBank and compiled a SCZ-specific adverse drug interaction network. This network included 28 SCZ drugs, 241 non-SCZs, and 991 interactions. By integrating the Anatomical Therapeutic Chemical (ATC) classification with the network analysis, we characterized those interactions. Our results indicated that SCZ drugs tended to have more adverse drug interactions than other drugs. Furthermore, SCZ typical drugs had significant interactions with drugs of the “alimentary tract and metabolism” category while SCZ atypical drugs had significant interactions with drugs of the categories “nervous system” and “antiinfectives for systemic uses.” This study is the first to characterize the adverse drug interactions in the course of SCZ treatment and might provide useful information for the future SCZ treatment.

## 1. Introduction

Schizophrenia (SCZ) is a common, complex mental disorder with a worldwide prevalence of approximately 1%, creating a substantial healthcare challenge in the world. Over the past several decades, antipsychotic drugs have been the commonly used medications to treat psychiatric disorders such as SCZ and bipolar disorder [1]. These drugs are classified as two types: typical and atypical antipsychotics. Typical antipsychotics are known as first generation antipsychotics, while atypical antipsychotics are known as second generation antipsychotics. Their significant difference lies in their different ability to produce extrapyramidal side effects (EPS), block dopamine type 2 receptors, improve negative symptoms, and others [2]. Antipsychotics can be effective in the treatment of SCZ but vary in efficacy and side effects. As the representative drugs of typical and atypical antipsychotics, respectively, haloperidol and clozapine are often utilized to characterize the effects of each of the two drug categories [2, 3]. In addition to the positive, negative, and cognitive symptoms, which are the hallmarks of psychotic illness, patients with schizophrenia are highly likely to have comorbid medical and other conditions such as anxiety disorders, depression, cardiovascular disease, and diabetes [4, 5]. Therefore, prescribed coadministration of antipsychotics and nonantipsychotics drugs has been increasing for the treatment of SCZ patients along with various aspects of their illness [6]. 

In healthcare, serious adverse effects have been reported in the coadministration of multiple drugs in many diseases such as heart disease [7], glaucoma [8], and cancer [9, 10]. It is estimated that approximately 20–30% of all adverse reactions are caused by interactions between drugs [11]. The adverse drug-drug interaction is defined as the phenomenon that occurs when the effects of a drug are altered by prior administration or coadministration of a second drug. It could happen during the drug absorption, the metabolism process, and the binding process of drug targets [12]. As the coadministration of multiple drugs to treat the psychiatric and nonpsychiatric comorbid medical conditions of schizophrenia increases, the potential of adverse drug-drug interactions (DDIs) is becoming an important consideration in the treatment of SCZ [6]. 

Numerous studies have focused on adverse drug-drug interactions associated with antipsychotics [13, 14]. However, few of them have comprehensively characterized the adverse interactions of these antipsychotics either among themselves or with non-SCZ drugs. Most of these studies focused on the collection of adverse interactions and their clinical characteristics [13, 14]. Additionally, for typical and atypical antipsychotics, various studies have been conducted to illustrate both adverse effects pinpointed in clinical trials and underlying molecular interactions between these two types of drugs and their targets [15–17]. However, the differences between their adverse interaction characteristics have never been investigated. These limitations were mainly attributed to an absence of comprehensive clinical and molecular data. These limitations have recently been largely eased thanks to a comprehensive and publicly available database DrugBank [18–20], a unique resource that combines detailed drug data. Thus, the ability to characterize the drug interactions of these antipsychotics is emerging; this in turn will provide a wide-ranging view of the drug-drug interactions of SCZ drugs and potential information for SCZ drug coadministration and prediction of adverse drug-drug interactions. 

In this study, we extracted 32 SCZ drugs based on the records in multiple fields from the DrugBank database and the suggestions from one of us (AHF), a practicing psychiatrist treating mostly psychotic disorders. We then collected the adverse drug-drug interactions of these SCZ drugs from the DrugBank to build an adverse drug-drug interaction network. Combing the network analysis with the drug classification from the Anatomical Therapeutic Chemical (ATC) systems [21], we characterized the adverse drug interactions of SCZ drugs as compared to other types of drugs. Additionally, we compared the properties of adverse interactions of SCZ typical and atypical drugs. This study represents the first to investigate the adverse drug interactions of SCZ drugs. The results may assist researchers to develop better diagnostic tests, effective medications, and coadministration strategies.

## 2. Materials and Methods

### 2.1. Collection of Drugs to Treat Schizophrenia

To collect a comprehensive list of the drugs that are routinely used to treat schizophrenia patients, we first utilized the schizophrenia related keywords, schizophrenia, schizophrenias, schizophrenic, schizophrenics, schizotypy, and schizotypal, to search the data from the DrugBank (version 3.0) [18]. The DrugBank contained 6796 drugs including 1571 approved drugs. Our search resulted in 46 drugs. Then, we checked the “indication” field, which describes common names of diseases that a drug is used to treat. We obtained 38 drugs that had the SCZ related keywords in the “indication” field and eight drugs that did not. Next, we accessed the DrugBank website and manually checked if each of these drugs has been used to treat schizophrenia. We found that five of these 38 drugs were not related to SCZ treatment. Thus, we obtained 32 SCZ drugs. Among them, 28 drugs have adverse drug-drug interactions according to the DrugBank. To obtain information regarding typical or atypical classification for these 28 drugs, we manually checked data from multiple resources: DrugBank, PubMed Health (http://www.ncbi.nlm.nih.gov/pubmedhealth/), Wikipedia (http://www.wikipedia.org/), and several textbooks.

### 2.2. Collection of Adverse Drug Interactions and Construction of Adverse Drug-Drug Interaction Networks

The DrugBank consists of adverse drug-drug interactions and represents the most complete, publicly accessible collection of its kind [19]. These adverse drug-drug interactions are the events that occur when the effects of a drug are altered by prior administration or coadministration of another drug. In DrugBank, for a given drug, the “interaction” field includes drugs and their corresponding adverse descriptions with the drug. These descriptions were compiled from a variety of web and textbook resources and verified by accredited pharmacists. We extracted all adverse drug interactions from the DrugBank data. Considering that the descriptions of adverse drug-drug interactions are very complicated, in this study, we formed a pair of drugs if both are involved in one adverse drug-drug interaction, but the direction of the interaction in each pair was ignored. Based on these drug pairs, we constructed an adverse drug interaction network as the human adverse DDI network, in which a node denotes a drug and an edge indicates that the two drugs would have some adverse events if they were coadministrated. From all adverse interactions, we extracted the adverse drug interactions of SCZ drugs including both interactions among SCZ drugs and interactions between SCZ drugs and non-SCZ drugs. Based on these SCZ drug interactions, we constructed a SCZ-specific DDI network, in which nodes are SCZ drugs and non-SCZ drugs and edges are the adverse interactions among SCZ drugs or the adverse interactions between SCZ drugs and non-SCZ drugs.

### 2.3. ATC Drug Classification

The World Health Organization (WHO) Collaborating Centre for Drug Statistics Methodology developed and maintains the Anatomical Therapeutic Chemical (ATC) classification database (http://www.whocc.no/). The classification system curates drugs into different groups according to their therapeutic, chemical, and pharmacological properties. It has five levels that represent progressively finer classifications. For example, the letter “N” represents the top level of the classification “nervous system.” The N class is further divided into, for example, N05 (psycholeptics) on the second level, N05A (antipsychotics) on the third level, N05AH (diazepines, oxazepines, thiazepines, and oxepines) on the fourth level, and N05AH02 (clozapine) on the fifth level. 

In this study, we applied the first-level classification to examine the general network properties of SCZ drugs. The first-level classification indicates the anatomical main group and consists of 14 main groups represented by 14 letters (codes). We further employed the third-level classification to examine the difference between classification of SCZ typical and atypical drugs. The third level classifies drugs based on mixed criteria involving therapeutic or pharmacological properties. We obtained drug ATC codes from DrugBank and the Kyoto Encyclopedia of Genes and Genomes (KEGG DRUG) database [22].

### 2.4. Network Topological Analysis and Visualization

In this study, we applied the node connectivity to examine the network topological property difference of SCZ drugs. For a given node in a network, node connectivity (degree) is the number of edges linked to the node, which is the network's most elementary characteristics [23]. Considering that SCZ drugs belong to the “nervous system” category, we compiled another two drug sets: other nervous system drugs (other N-drugs) and drugs excluding nervous systems drugs (non-N-drugs); we then compared their degree distributions in the context of all human adverse DDIs. We utilized the software Cytoscape for network visualization [24]. 

### 2.5. Statistical Tests

We employed the Wilcoxon rank-sum test to compare degree distribution. To compare adverse interaction tendencies of SCZ typical and atypical drugs, we divided the SCZ drug interactions into 14 categories according to their linked non-SCZ drugs' ATC first-level classification, and then we performed Fisher's exact test for each category. For a given category, we calculated a 2 × 2 contingency table, which includes four counts: *n*, *N*−*n*, *r*, and *R*−*r*, where *n* is the count of the links that SCZ typical drugs have in the category, *N* is the count of total links that SCZ typical drugs have in all 14 categories, *r* is the count of the links that SCZ atypical drugs have in the category, and *R* is the count of total links that SCZ atypical drugs have in all 14 categories. We utilized the R package (http://www.r-project.org/) to calculate *P*values followed by multiple testing correction using the Bonferroni method [25].

## 3. Results

In this study, we collected 32 drugs that are mainly used to treat the schizophrenia patients, denoted as SCZ drugs. Among them, 28 drugs had adverse drug-drug interactions according to DrugBank data. Within this list, 18 SCZ drugs belonged to typical antipsychotics while the other 10 belonged to atypical antipsychotics category (Table 1). To explore the characteristics of SCZ adverse drug interactions, we first collected all the adverse DDIs from DrugBank and constructed a human DDI network, which included 10,931 pairs involving 1087 drugs. Among these 1087 drugs, 1005 had at least one ATC annotation, and they were classified into 14 drug sets based on their ATC first-level classification. 

### 3.1. SCZ Drugs Had a Significant Higher Degree of Adverse Interactions Than Other Drugs

**Figure 1:**
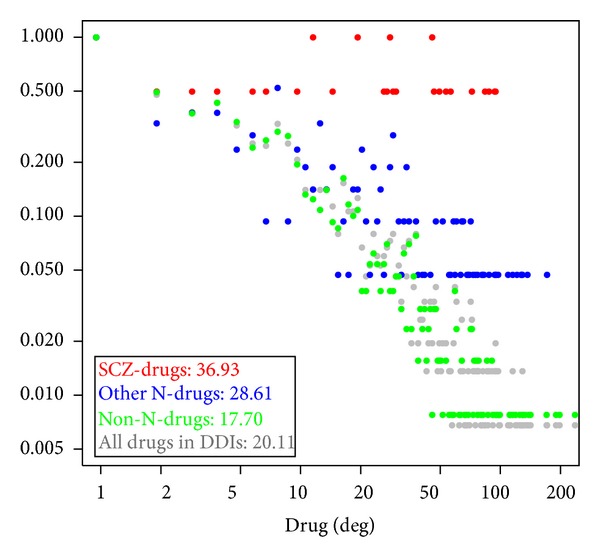
Degree distributions and average degrees (vertical lines) of four drug sets. *Y*-axis represents the proportion of drugs that have a degree while the *X*-axis is the drug degree. “SCZ-drugs” (red) denotes the 28 schizophrenia (SCZ) drugs. “other N-drugs” (blue) denotes the drugs belonging to the “nervous systems” category after exclusion of SCZ drugs. “non-N-drugs” (green) denotes the drugs not belonging to “nervous systems.” “all drugs in DDIs” (grey) denotes all drugs in the human DDIs. The inserted table summarizes the average degree for each drug set.

For comparison, we compiled three drug sets based on all drugs in human DDIs: SCZ drugs, other N-drugs, and non-N-drugs. Then, we calculated degree distributions and average degrees for three drug sets and all drugs in DDIs.  Figure 1 displays their degree distributions and average degrees. The average degree of SCZ drugs was 36.93, which was significantly higher than that of other N-drugs (28.61, Wilcoxon's test, *P* value = 0.0209) or that of non-N-drugs (17.70, *P* value = 6.45 × 10^−6^). This observation indicated that, compared to non-SCZ drugs, SCZ drugs tended to have more adverse drug interactions with other drugs. Moreover, other N-drugs had significantly more adverse drug interactions than that of the non-N-drugs (*P* value = 5.98 × 10^−7^). This observation indicated that drugs belonging to the “nervous systems” category tended to have more adverse drug interactions with other drugs.

To explore this tendency in detail, we examined if the N-drug set is different from the other 13 drug categories in all DDIs based on the ATC first-level annotation. According to the ATC first-level annotation, 1005 drugs in DDIs with at least one ATC drug annotation could be grouped into 14 groups. Among the 1005 drugs, those with multiple ATC codes were assigned to multiple groups. We performed Wilcoxon's rank sum test to examine if the degree distribution of the N-drugs is different from that of each of the other 13 groups of drugs. We found that N-drugs had significantly more adverse drug interactions than the other eight groups (*P* value < 0.05) (Table 2). These other eight groups were “antineoplastic and immunomodulating agents” (L), “various” (V), “alimentary tract and metabolism” (A), “blood and blood forming organs” (B), “sensory organs” (S), “respiratory system” (R), “anti-infectives for systemic use” (J), and “musculoskeletal system” (M). Notably, among the eight groups, four passed the stringent Bonferroni multiple testing correction (Bonferroni adjusted *P*-value < 0.05). These four were “antineoplastic and immunomodulating agents” (L), “various” (V), “alimentary tract and metabolism” (A), and “blood and blood forming organs” (B). These results indicated that the N-drugs had different adverse interaction tendencies compared to other groups of drugs; specifically their tendencies were different from the drugs in groups L, V, A, and B.

### 3.2. SCZ Adverse DDI Network

**Figure 2:**
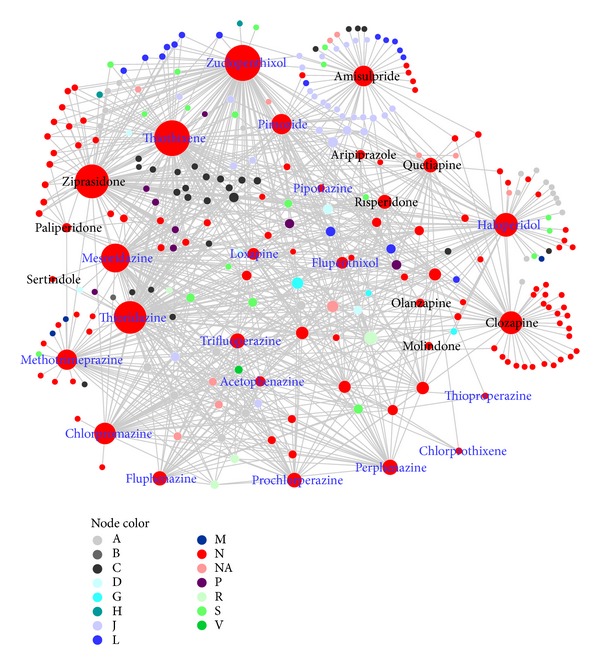
Adverse drug-drug interaction network for schizophrenia (SCZ) drugs. Node color corresponds to ATC first-level classification code. With the exception of “NA” for the drugs without ATC classification, the representations of these letters are detailed in Table 2. Nodes with blue labels are SCZ typical drugs, and nodes with black labels are SCZ atypical drugs. Node size corresponds to the number of the adverse interactions that the drug had in the network.

From the human DDIs compiled in this study, 28 SCZ drugs had 991 interactions in total, in which 43 interactions occurred among SCZ drugs and 948 interactions occurred between SCZ drugs and non-SCZ drugs (Figure 2). The average degree of the 28 drugs was 36.93. Among these SCZ drugs, 11 drugs had more than forty interactions. These 11 drugs were zuclopenthixol (degree: 97), thiothixene (96), ziprasidone (90), thioridazine (86), mesoridazine (74), haloperidol (58), clozapine (55), chlorpromazine (51), amisulpride (48), methotrimeprazine (47), and pimozide (47). Among the 11 drugs, three were atypical drugs: clozapine, ziprasidone, and amisulpride. The average degree of the 241 non-SCZ drugs was 3.92. Among these 241 drugs, 22 drugs had more than 10 interactions with SCZ drugs. They were triprolidine (23), tacrine (22), tetrabenazine (22), galantamine (21), donepezil (21), trospium (19), trimethobenzamide (19), voriconazole (15), rivastigmine (15), tacrolimus (13), bromocriptine (13), vorinostat (13), lumefantrine (13), nilvadipine (12), toremifene (12), cisapride (12), trimipramine (12), guanethidine (12), artemether (12), desvenlafaxine (12), levofloxacin (11), and sparfloxacin (11).

Among 991 interactions, 43 interactions occurred among 16 SCZ drugs, while 948 occurred between 28 SCZ drugs and 241 non-SCZ drugs. According to the description of the 43 adverse drug interactions, 41 were related to increased risk of cardiotoxicity and arrhythmias, which indicated that the coadministration of SCZ drugs might increase the risk of heart disease, especially for prolonging QT intervals [26]. 

Since all of the 28 SCZ drugs involved in the 991 interactions belonged to the “nervous systems” category, we grouped the 991 interactions into 14 categories based on their interacting drugs' ATC first-level annotation. Among the 241 drugs that had interactions with 28 SCZ drugs, 229 drugs had an ATC annotation. Based on these drug ATC first-level codes, we categorized the interactions into 14 categories (Additional file, Table S1 available online at http://dx.doi.org/10.1155/2013/458989). Among them, the top 3 types of interactions are “nervous system” (N), “anti-infectives for systemic use” (J), and “cardiovascular system” (C). This observation indicated that SCZ drugs tended to have adverse interactions with these three types of non-SCZ drugs.

### 3.3. Comparison of SCZ Typical and Atypical Drugs

Among the 28 SCZ drugs with adverse drug-drug interactions, 18 were typical antipsychotic drugs and 10 were atypical antipsychotic drugs. The average degree of typical drugs in the SCZ DDI network was 41.22, while that of atypical drugs was 29.20. The former was much higher than the later, indicating that typical drugs had more adverse interactions than atypical drugs.

Among the 948 interactions between 28 SCZ drugs and 241 non-SCZ drugs, 680 of the interactions occurred between 18 typical drugs and 188 non-SCZ drugs while the other 268 occurred between 10 atypical drugs and 164 non-SCZ drugs. To examine the difference in adverse drug interactions between typical and atypical drugs, we divided these interactions into 14 groups according to their interaction with non-SCZ drugs' ATC first-level classifications. Among the 241 non-SCZ drugs, 230 drugs had at least one ATC annotation. For the 680 typical SCZ drug interactions, 638 could be grouped into 14 groups, and for the 268 atypical SCZ drug interactions, 258 could be grouped into 11 groups. We performed Fisher's exact test for each group (Table 3). These results showed that SCZ typical and atypical drugs had significant differences at three ATC first-level categories: “alimentary tract and metabolism” (A), “nervous system” (N), and “anti-infectives for systemic use” (J). More specifically, for the category “alimentary tract and metabolism” (A), the interaction number and percentage of typical SCZ drugs were higher than those of SCZ atypical drugs. In contrast, for the categories “nervous system” (N) and “anti-infectives for systemic use” (J), the interaction percentage of SCZ atypical drugs was higher than that of SCZ typical drugs. These observations revealed that SCZ typical drugs had significant interactions with drugs belonging to the category “alimentary tract and metabolism” (A), while SCZ atypical drugs tended to have more interactions with drugs belonging to the categories “nervous system” (N) and “anti-infectives for systemic use” (J). 

To further interrogate the detailed difference between the two SCZ drug categories, we performed the comparison of adverse interactions between SCZ typical and atypical drugs using the ATC third-level classification. According to the third-level classification, among the 680 adverse interactions of 18 SCZ typical drugs, 637 could be categorized into 57 third-level categories while the remaining 43 interactions could not be similarly classified due to a lack of ATC annotation for interacting non-SCZ drugs. Similarly, among 268 adverse interactions of 10 SCZ atypical drugs, 251 adverse interactions could be sorted into 44 third-level categories while the other 17 interactions could not. Among these 57 and 44 third-level categories, there were 39 common categories in both SCZ typical and atypical drug adverse interactions sets, 18 categories were specific for SCZ typical drugs, and 5 were specific to SCZ atypical drugs (Additional file, Table S2). Among these categories that are specific for typical and atypical drugs, most of them had only a few interactions, except for categories “antiobesity preparations, excluding diet products” (A08A), and “antiglaucoma preparations and miotics” (S01E). SCZ typical drugs had 45 interactions (7.06%) in A08A and 13 interactions (2.04%) in S01E while SCZ atypical drugs had none in either category. The category A08A included antiobesity drugs excluding diet products while S01E included the drugs used to treat glaucoma and related diseases. The observation may indicate that, compared to atypical drugs, typical drugs tended to have adverse drug-drug interactions with antiobesity drugs and antiglaucoma drugs.

Among the 39 common categories of SCZ drug adverse interactions, seven had significant difference between typical and atypical drugs (Fisher's exact test, *P* value < 0.05, Table 4). Among them, four belonged to the category “nervous systems” (N) and the other three belonged to the category “anti-infectives for systemic use” (J). This is consistent with the above observations. For the categories “psychostimulants, agents used for ADHD and nootropics” (N06B), and “quinolone antibacterials” (J01M), the percentage of SCZ typical drug adverse interactions was significantly higher than that of SCZ atypical drug adverse interactions, indicating that SCZ typical drugs tended to have adverse interactions with these drugs belonging to the N06B and J01M categories. On the contrary, the percentage of SCZ atypical drug adverse interactions was significantly higher than those of SCZ typical drug adverse interactions in the categories “antiepileptics” (N03A), “anxiolytics” (N05B), “hypnotics and sedatives” (N05C), and “drugs for treatment of tuberculosis” (J04A). This result suggested that SCZ atypical drugs might have adverse interactions with these drugs belonging to the categories N03A, N05B, N05C, and J04A.

### 3.4. Clozapine Tended to Have More Adverse Interactions with Non-N-Drugs but Not with SCZ Drug

**Figure 3:**
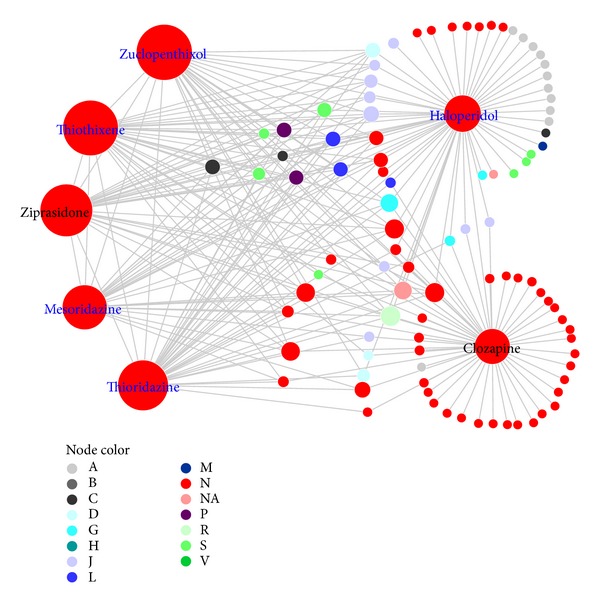
Haloperidol and clozapine adverse drug-drug interaction subnetwork extracted from the schizophrenia (SCZ) adverse drug-drug interaction network. Node color corresponds to ATC first-level classification code. With the exception of “NA” for the drugs without ATC classification, the representations of these letters are detailed in Table 2. Nodes with blue labels are SCZ typical drugs, and nodes with black labels are SCZ atypical drugs. Node size corresponds to the number of adverse interactions that the drug had in the SCZ drug-drug interaction network.

Haloperidol and clozapine are the representative drugs for SCZ typical and atypical drugs, respectively. Haloperidol had adverse interactions with 58 drugs while clozapine had adverse interactions with 55 drugs.  Figure 3 showed the merged subnetwork for haloperidol and clozapine adverse drug-drug interactions. Among the 58 drugs having adverse interactions with haloperidol, 27 (46.55%) belonged to the “nervous system” category, while among the 55 drugs that have adverse interactions with clozapine, 40 (72.73%) belonged to the same category. The results showed that haloperidol might tend to have adverse drug interactions with non-N-drugs while clozapine might tend to have adverse drug interactions with N-drugs. However, in contrast to clozapine, haloperidol had adverse interactions with five other SCZ drugs (mesoridazine, thioridazine, thiothixene, ziprasidone, and zuclopenthixol) while clozapine had only adverse interactions with haloperidol (Figure 3 and Additional file, Figure S1). This observation revealed that, for SCZ drugs, haloperidol had adverse interactions with other SCZ drugs but clozapine did not.

## 4. Discussion

In this study, we began with a comprehensive compilation of schizophrenia (SCZ) drugs and their adverse drug-drug interactions, and then we performed comprehensive comparisons between SCZ drugs and other types of drugs as well as SCZ typical and atypical antipsychotics. The results in this study had shown that SCZ drugs had different adverse interaction tendencies, and these differences extended to SCZ typical and atypical drugs, as well. This study provides the global view of the characteristics of antipsychotics adverse interactions either among themselves or with non-SCZ drugs. Additionally, this study might provide potential adverse drug-drug interactions between SCZ drugs and non-SCZ drugs, which have never been used to treat SCZ or have no records of adverse drug-drug interactions. If these non-SCZ drugs have ATC classification annotation, physicians may consider whether the characteristics of non-SCZ drug categories have a higher probability to cause adverse drug-drug interactions with SCZ drugs. Thus, before prescribing these drugs belonging to these categories to SCZ patients, physicians would be more cautious.

One important output of this study was that, compared to other types of drugs, the drugs belonging to the “nervous system” (N) tended to have more adverse drug interactions, especially SCZ drugs. This was not surprising since it is well known that SCZ drugs affect neurotransmitter systems that are common to psychotropic medications used to treat other disorders. For example, while atypical antipsychotics block the dopamine and serotonin receptors, most antidepressants increase serotonergic levels while others increase levels of both serotonin and dopamine. Furthermore, many antidepressants as well as antipsychotics have known anticholinergic properties.

During the past several decades, numerous studies have revealed the difference in side effects between typical and atypical antipsychotics. However, the mechanisms underlying this difference are unclear. To characterize the effects of each of the two drug categories, researchers often utilize two drugs, haloperidol and clozapine, as the representatives of typical and atypical antipsychotics. In this study, we observed that haloperidol tended to have adverse interactions with other SCZ drugs but this is not the case for clozapine. This observation might suggest that their functional pathways in their drug action be different. Therefore, understanding the molecular mechanisms underlying those drug actions is critical for developing effective diagnostic tests and medications. Neurotransmitter receptors are primary targets of antipsychotics, and their interactions are important for drug efficacy. However, additional therapeutic properties may not directly relate to the receptor mechanism but to the intracellular signaling cascades. Through these cascades, the chemical signals of drug receptor interactions transfer to alter gene expression, further affecting the formation of phenotypes. Therefore, future research endeavors may ensure investigating the post-receptor mechanisms of antipsychotics.

We mainly extracted the adverse drug-drug interaction data from DrugBank for this study. Though the study provides a review of the adverse drug-drug interactions of SCZ drugs, this investigation still needs to be improved since the current data utilized here is neither complete nor bias-free. Thus, future research in this area should include more adverse drug interaction data from multiple databases and other text resources such as Drug Interaction Facts [12], Drug Interaction Analysis and Management [27], Micromedex Drug-REAX [28], and the KEGG DRUG database [22]. Additionally, as a large volume of electronic medical records (EMRs) and FDA drug labels become available and effective text mining approaches were developed [29], the development of the novel methods to integrate multidimensional data sources and build a comprehensive resource for adverse drug interactions will be possible and practical. 

Additionally, DDIs can occur through several biological processes of drug disposition, which might affect the same targets, the same metabolic pathways, or the same signaling pathways [30]. Considering the limitation of current molecular data, in this study, we did not explore the molecular mechanisms underlying the adverse drug interactions of SCZ drugs. However, a large volume of genome-wide molecular neuropharmacology data, such as microarray gene expression [31] and genome-wide association studies [32], is available, and more large-scale data will be available in the near future due to the rapid advances in genome-wide technologies and strong support from pharmacology communities. Therefore, it is possible and necessary to develop novel detection methods for investigation of adverse DDIs based on the molecular data. This will not only provide the valuable information for physicians, but also create a deeper understanding of the molecular mechanisms underlying adverse drug-drug interaction side effects, thereby furthering the ability to detect potential drug interactions. 

## 5. Conclusions

In this study, we presented a comprehensive investigation of adverse drug-drug interactions of the antipsychotics used to treat schizophrenia. We integrated the network analysis with ATC drug classifications, which provided the adverse drug interaction characteristics of SCZ drugs as well as typical and atypical drugs. However, much more work is needed to collect more adverse drug interaction information and develop advanced pharmacogenomics network approaches. Potential findings could be used to predict adverse drug-drug interactions and improve the coadministration of multiple drugs, which in turn may lead to the avoidance of the drug-drug interaction adverse effects.

## Supplementary Material

The Supplementary Materials include two tables and one figure. Table S1 includes the adverse interaction categories of schizophrenia (SCZ) drugs based on the ATC first-level classification. Table S2 includes the adverse interaction categories of SCZ drugs according to ATC third-level classification. Figure S1 shows adverse drug-drug interactions among SCZ drugs.

## Figures and Tables

**Table 1:** Antipsychotics used to treat schizophrenia patients.

DrugBank ID	Drug name	Number of adverse drug interactions	Typical/atypical^a^
DB01063	Acetophenazine	15	Typical
DB06288	Amisulpride	48	Atypical
DB01238	Aripiprazole	12	Atypical
DB00477	Chlorpromazine	51	Typical
DB01239	Chlorprothixene	4	Typical
DB00363	Clozapine	55	Atypical
DB00875	Flupenthixol	20	Typical
DB00623	Fluphenazine	28	Typical
DB00502	Haloperidol	58	Typical
DB00408	Loxapine	20	Typical
DB00933	Mesoridazine	74	Typical
DB01403	Methotrimeprazine	47	Typical
DB01618	Molindone	7	Atypical
DB00334	Olanzapine	10	Atypical
DB01267	Paliperidone	12	Atypical
DB00850	Perphenazine	31	Typical
DB01100	Pimozide	47	Typical
DB01621	Pipotiazine	6	Typical
DB00433	Prochlorperazine	29	Typical
DB01224	Quetiapine	29	Atypical
DB00734	Risperidone	27	Atypical
DB06144	Sertindole	2	Atypical
DB01622	Thioproperazine	3	Typical
DB00679	Thioridazine	86	Typical
DB01623	Thiothixene	96	Typical
DB00831	Trifluoperazine	30	Typical
DB00246	Ziprasidone	90	Atypical
DB01624	Zuclopenthixol	97	Typical

^a^Antipsychotic drugs are classified as typical and atypical mainly based on their different ability to cause extrapyramidal side effects (EPS).

**Table 2:** Comparison of drugs belonging to the “nervous Systems” category with drugs from the other categories based on their degrees in human adverse drug-drug interaction network.

ATC first-level classification (ATC code)	Number of drugs	Average degree	Wilcoxon test *P*-value	Bonferroni adjusted *P*-value
Antineoplastic and immunomodulating agents (L)	118	15.25	3.01 × 10^−8^	4.22 × 10^−7^
Various (V)	22	7.14	5.03 × 10^−6^	7.04 × 10^−5^
Alimentary tract and metabolism (A)	119	15.52	1.49 × 10^−5^	2.09 × 10^−4^
Blood and blood forming organs (B)	45	21.00	3.02 × 10^−4^	0.0042
Sensory organs (S)	85	19.66	0.0089	0.1242
Respiratory system (R)	75	20.27	0.0148	0.2068
Antiinfectives for systemic use (J)	143	25.19	0.0228	0.3186
Musculoskeletal system (M)	56	14.39	0.0287	0.4021
Cardiovascular system (C)	153	22.75	0.7008	1.000
Dermatologicals (D)	56	25.27	0.4775	1.000
Genitourinary system and sex hormones (G)	61	21.38	0.1570	1.000
Systemic hormonal preparations, excluding sex hormones and insulins (H)	21	20.76	0.5294	1.000
Antiparasitic products, insecticides, and repellents (P)	20	19.95	0.2517	1.000
Nervous system (N)	219	29.68	—	—

**Table 3:** Comparison of interaction categories between typical and atypical SCZ drugs using ATC first-level classification.

ATC first-level classification (ATC code)	Number of typical drug interactions (%^a^)	Number of atypical drug interactions (%^b^)	Fisher's exact test *P*-value
Alimentary tract and metabolism (A)	74 (11.60)	5 (1.94)	3.94 × 10^−7^
Nervous system (N)	216 (33.86)	119 (46.12)	0.0008
Antiinfectives for systemic use (J)	66 (10.34)	46 (17.83)	0.0035
Sensory organs (S)	51 (7.99)	12 (4.65)	0.0839
Various (V)	8 (1.25)	0 (0)	0.1136
Respiratory system (R)	35 (5.49)	8 (3.10)	0.1668
Antineoplastic and immunomodulating agents (L)	37 (5.80)	21 (8.14)	0.2297
Antiparasitic products, insecticides, and repellents (P)	36 (5.64)	10 (3.88)	0.3191
Cardiovascular system (C)	68 (10.66)	23 (8.91)	0.4662
Musculoskeletal system (M)	3 (0.47)	0 (0)	0.5612
Genitourinary system and sex hormones (G)	19 (2.98)	6 (2.33)	0.6618
Dermatologicals (D)	21 (3.29)	7 (2.71)	0.8325
Systemic hormonal preparations, excluding sex hormones and insulins (H)	3 (0.47)	1 (0.39)	1.0000
Blood and blood forming organs (B)	1 (0.16)	0 (0)	1.0000

^a^The percentage was calculated by the number of interactions with SCZ atypical in each category divided by all the numbers of interactions with SCZ atypical drugs.

^b^The percentage was calculated by the number of interactions with SCZ typical in each category divided by all the numbers of interactions with SCZ typical drugs.

**Table 4:** Comparison of interaction categories between SCZ typical and atypical drugs using ATC third-level classification.

ATC third-level classification (ATC code)	Number of typical drug interactions (%^a^)	Number of atypical drug interactions (%^b^)	Fisher's exact test *P*-value
Antiepileptics (N03A)	3 (0.47)	21 (3.30)	1.95 × 10^−8^
Anxiolytics (N05B)	1 (0.16)	11 (1.73)	2.91 × 10^−5^
Hypnotics and sedatives (N05C)	3 (0.47)	10 (1.57)	1.13 × 10^−3^
Psychostimulants, agents used for ADHD and nootropics (N06B)	28 (4.40)	2 (0.31)	0.0012
Direct acting antivirals (J05A)	13 (2.04)	17 (2.67)	0.0051
Quinolone antibacterials (J01M)	18 (2.83)	1 (0.16)	0.0107
Drugs for treatment of tuberculosis (J04A)	2 (0.31)	6 (0.94)	0.0161

^a^The percentage was calculated by the number of interactions with SCZ atypical in each category divided by all the numbers of interactions with SCZ atypical drugs.

^b^The percentage was calculated by the number of interactions with SCZ typical in each category divided by all the numbers of interactions with SCZ typical drugs.
