# Supplementary material for: Characterization of Schizophrenia Adverse Drug Interactions through a Network Approach and Drug Classification
Source: Biomed Res Int. 2013 Sep 9;2013:458989. doi: 10.1155/2013/458989 (PMC3782118; doi:10.1155/2013/458989)
Supplement: Supplementary file 1 — The Supplementary Materials include two tables and one figure. Table S1 includes the adverse interaction categories of schizophrenia (SCZ) drugs based on the ATC first-level classification. Table S2 includes the adverse interaction categories of SCZ drugs according to ATC third-level classification. Figure S1 shows adverse drug-drug interactions among SCZ drugs. [file 458989.f1.pdf]

Additional file

TABLE S1. Adverse Interaction Categories of SCZ Drugs Based on the ATC First-level Classification

**Interaction Category**<br><br>**(ATC First-level Classification)**	**Number of Non-SCZ Drugs**	**Number of Interactions**	**Percentage of Drugs**
Nervous system (N)	92	339	0.3294
Antiinfectives for systemic use (J)	29	157	0.1526
Cardiovascular system (C)	26	105	0.1020
Alimentary tract and metabolism (A)	22	82	0.0797
Antineoplastic and immunomodulating agents (L)	15	68	0.0661
Sensory organs (S)	18	63	0.0612
Dermatologicals (D)	5	51	0.0496
Genito-urinary system and sex hormones (G)	3	50	0.0486
Antiparasitic products, insecticides and repellents (P)	8	49	0.0476
Respiratory system (R)	4	48	0.0466
Various (V)	1	8	0.0078
Systemic hormonal preparations, excluding sex hormones and insulins (H)	2	5	0.0049
Musculo-skeletal system (M)	3	3	0.0029
Blood and blood forming organs (B)	1	1	0.0010

Table S2. Adverse Interaction Categories of SCZ Drugs According to ATC Third-level Classification

ATC Code	Number of Interactions of Typical SCZ Drugs	Percentage of Typical SCZ Drugs Interactions (%)	Number of Interactions of Atypical SCZ Drugs	Percentage of Atypical SCZ Drugs Interactions (%)
A02B	0	0	1	0.16
C03X	0	0	1	0.16
C09A	0	0	1	0.16
D07A	0	0	1	0.16
L01B	0	0	2	0.31
A03B	1	0.16	0	0
B01A	1	0.16	0	0
C02A	1	0.16	0	0
D05B	1	0.16	0	0
H05B	1	0.16	0	0
M03C	1	0.16	0	0
N05A	1	0.16	0	0
A10B	2	0.31	0	0
C08D	2	0.31	0	0
M03B	2	0.31	0	0
R02A	2	0.31	0	0
S01F	3	0.47	0	0
N04A	5	0.78	0	0

A03A	8	1.26	0	0
V08A	8	1.26	0	0
R01B	9	1.41	0	0
S01E	13	2.04	0	0
A08A	45	7.06	0	0
C02K	1	0.16	1	0.16
S03A	1	0.16	1	0.16
N05B	1	0.16	11	1.73
C03B	2	0.31	1	0.16
C10A	2	0.31	1	0.16
H01C	2	0.31	1	0.16
L03A	2	0.31	1	0.16
N01A	2	0.31	1	0.16
N07B	2	0.31	1	0.16
J04A	2	0.31	6	0.94
A04A	3	0.47	1	0.16
C01E	3	0.47	1	0.16
G04C	3	0.47	1	0.16
G01A	3	0.47	5	0.78
N05C	3	0.47	10	1.57
N03A	3	0.47	21	3.30
P01C	4	0.63	1	0.16
N02A	5	0.78	1	0.16
L04A	9	1.41	5	0.78
C07A	11	1.73	1	0.16

C08C	12	1.88	5	0.78
D01A	13	2.04	4	0.63
J01F	13	2.04	9	1.41
J02A	13	2.04	9	1.41
J05A	13	2.04	17	2.67
A03F	14	2.20	3	0.47
L02B	14	2.20	6	0.94
N07X	14	2.20	8	1.26
G04B	16	2.51	5	0.78
J01M	18	2.83	1	0.16
L01X	21	3.30	11	1.73
N04B	25	3.92	14	2.20
N06B	28	4.40	2	0.31
R06A	28	4.40	8	1.26
P01B	32	5.02	9	1.41
S01A	32	5.02	9	1.41
C01B	34	5.34	11	1.73
N06D	56	8.79	23	3.61
N06A	71	11.15	27	4.24

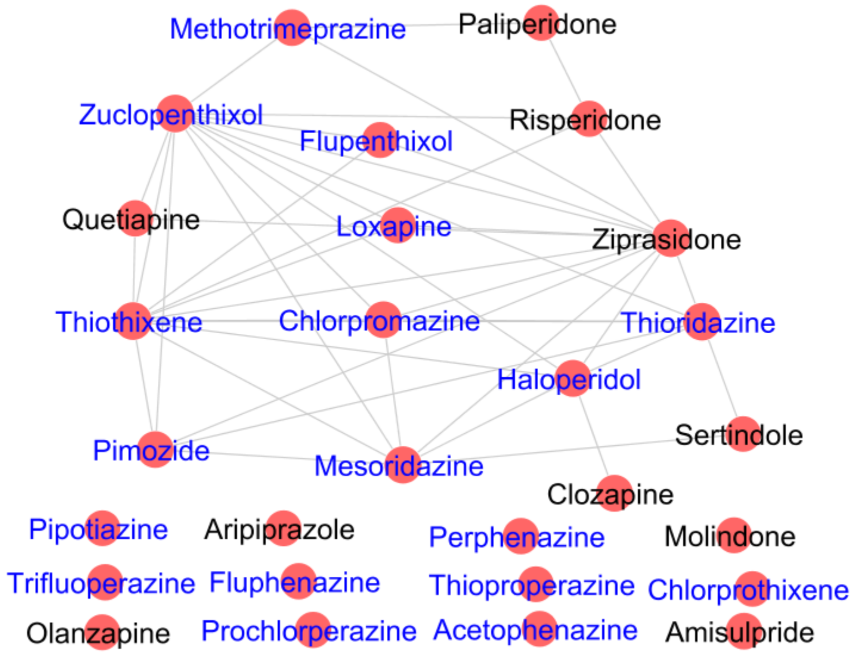

Figure S1. Adverse drug-drug interactions among SCZ drugs. Nodes with blue lables are SCZ typical drugs, and nodes with black lables are SCZ atypical drugs.
